# Preoperative medial knee instability is an underestimated risk factor for failure of revision ACL reconstruction

**DOI:** 10.1007/s00167-020-06133-y

**Published:** 2020-07-03

**Authors:** Lena Alm, Matthias Krause, Karl-Heinz Frosch, Ralph Akoto

**Affiliations:** 1 Department of Trauma and Orthopaedic Surgery, Sports Traumatology, BG Hospital Hamburg, Bergedorfer Str. 10, 21033 Hamburg, Germany; 2 Department of Trauma and Orthopaedic Surgery, University Medical Center Hamburg-Eppendorf, Martinistrasse 52, 20246 Hamburg, Germany; 3 Asklepios Clinic St. Georg, Department of Trauma and Reconstructive Surgery with Division of Knee and Shoulder Surgery, Sports Traumatology, Hamburg, Germany; 4 Department of Orthopaedics, Trauma Surgery and Sports Medicine, Cologne Merheim Medical Center, University of Witten/Herdecke, Cologne, Germany

## Abstract

**Purpose:**

The purpose of this study was to carefully analyse the reasons for revision ACLR failure to optimize the surgical revision technique and minimize the risk of recurrent re-rupture. Large studies with a minimum of 2 years of follow-up that clinically examine patients with revision ACLR are rare.

**Methods:**

Between 2013 and 2016, 111 patients who underwent revision ACLR were included in the retrospective study. All patients were examined for a minimum of 2 years after revision surgery (35 ± 3.4 months, mean ± STD) and identified as “failed revision ACLR” (side-to-side difference ≥ 5 mm and pivot-shift grade 2/3) or “stable revision ACLR”.

**Results:**

Failure after revision ACLR occurred in 14.5% (*n* = 16) of the cases. Preoperative medial knee instability (*n* = 36) was associated with failure; thus, patients had a 17 times greater risk of failure when medial knee instability was diagnosed (*p* = 0.015). The risk of failure was reduced when patients had medial stabilization (*n* = 24, *p* = 0.034) and extra-articular lateral tenodesis during revision surgery (*n* = 51, *p* = 0.028). Increased posterior tibial slope (*n* = 11 ≥ 12°, *p* = 0.046) and high-grade anterior knee laxity (side-to-side difference > 6 mm and pivot-shift grade 3, *n* = 41, *p* = 0.034) were associated with increased failure of revision ACLR. Obese patients had a 9 times greater risk of failure (*p* = 0.008, *n* = 30).

**Conclusion:**

This study demonstrates the largest revision ACLR patient group with pre- and postoperative clinical examination data and a follow-up of 2 years published to date. Preoperative medial knee instability is an underestimated risk factor for revision ACLR failure. Additionally, high-grade anterior knee laxity, increased PTS and high BMI are risk factors for failure of revision ACLR, while additional medial stabilization and lateral extra-articular tenodesis reduce the risk of failure.

**Level of evidence:**

III.

## Introduction

Results following revision anterior cruciate ligament reconstruction (ACLR) are commonly known to be less favourable than those following primary ACLR, as failure rates of 3.5–33% for revision and 5–25% for primary ACLR have been reported [9]. Reasons for failure of revision surgery are multifactorial. In the literature, the most common causes of failure are technical errors such as femoral tunnel malpositioning and trauma, followed by biological factors, untreated secondary instabilities and knee infection [12, 38, 43].

In recent years, the importance of secondary stabilizers in ACLR has been underestimated.

Concomitant lesions of the anterolateral ligament (ALL) can lead to recurrent instability after primary ACLR, and injury of the medial collateral ligament (MCL) complex is associated with a 13 times greater risk of failure [1, 34]. Accordingly, Louis et al. showed a reduced risk of failure when lateral extra-articular tenodesis was performed during revision surgery [25].

Patients have an increased risk of revision when the posterior tibial slope (PTS) is elevated or when high-grade anterior knee instability is present [5] [26]. The patient’s lifestyle can also affect the outcome of ACLR, as obesity can lead to a significantly increased risk of failure of ACLR [16, 36, 39].

A thorough analysis of the reasons for revision ACLR failure and a detailed preoperative clinical evaluation are essential to select the optimal technique for surgical revision.

Currently, there are a limited number of studies that have analysed failure after revision ACLR with a population of over 100 patients with a clinical postoperative examination, a minimum of 2 years of follow-up and a clear definition of revision ACLR failure. Diamantopoulos et al. presented the outcomes of 107 patients with revision ACLR and with a mean follow-up of 72.9 ± 20.6 months, but no preoperative clinical assessment was included in the study [7].

There is clear evidence that preoperative knee instability is associated with ACLR failure. Thus, Magnussen et al. showed that high-grade anterior knee instability, defined as International Knee Documentation Committee (IKDC) grade D in the Lachman, anterior drawer or pivot-shift tests, was associated with a significantly increased risk of ACLR failure [27]. Noyes et al. Salmon et al. and Yoon et al. presented clinical results of patients with revision ACLR that also included preoperative knee joint stability. However, none of these studies analysed the correlation between preoperative knee joint stability and revision ACLR failure [28, 32, 44].

To our knowledge, there is no study that directly relates the risk of failure of revision ACLR to preoperative instability of the knee.

The aim of this study was to perform a failure analysis of revision ACLR that encompasses preoperative knee instability in one of the largest evaluated populations of 111 patients with revision ACLR and with a minimum of 2 years of follow-up published to date.

It was hypothesized that the underestimated factor of preoperative medial knee instability is a risk for failure of revision ACLR and should be addressed at the time of revision surgery. Additionally, high-grade anterior knee instability, increased PTS and high BMI are risk factors for revision ACLR.

## Materials and methods

The study design was approved by the local ethics committee, and informed consent was obtained from each patient in the study (PV5590, Ärztekammer Hamburg). Between 2013 and 2016, all patients with revision ACLR were included in the cohort study (Table 1). A total of 135 patients after revision ACLR were eligible for the retrospective study, and 24 patients were excluded: 13 patients were lost to follow-up, another 7 suffered from knee infection preoperatively, and 4 also had an additional posterior cruciate ligament injury (Table 2).

**Table 1:** Selection process of the analysed patients with revision ACLR

	Number of patients (*n*)
Patients with revision ACLR between 2013 and 2016	135
Patients lost to follow-up	13
Patients excluded	11
Analysed patients	= 111

**Table 2:** Inclusion and exclusion criteria

Inclusion criteria	Exclusion criteria
Patients with revision ACLR operated on between 2013 and 2016	Patients who were lost to follow-up
Patients who agreed to participate in the study	Knee infection
	Additional posterior cruciate ligament injuries
	Osteoarthritis Kellgren–Lawrence grade 4

Subsequently, 111 patients with revision ACLR (43 women and 68 men, mean age of 30.1 ± 12.2 years (mean age ± STD), range 18–52 years) were examined with a follow-up of at least 2 years (35 ± 3.4 months, mean ± STD). Among them, patients were identified as “failed revision ACLR” (side-to-side difference ≥ 5 mm in Rolimeter® testing and/or pivot-shift grade 2/3) or “stable revision ACLR” [10].

### Clinical testing protocol before revision ACLR

Anterior translation of the tibia was measured clinically with the Lachman and pivot-shift tests and was objectively tested with the Rolimeter®. While the pivot-shift test was divided into grade 1 (glide), grade 2 (clunk) and grade 3 (gross), the Lachman test was measured with the 2000 IKDC Knee Examination Form (grade 1: 2–5 mm, grade 2: 6–10 mm and grade 3: > 10 mm) [20]. Medial and lateral knee instability was assessed clinically according to Hughston and the American Medical Association (AMA) [2, 18] [19]. Preoperatively, a high-grade anterior knee laxity was defined as a side-to-side difference greater than 6 mm and/or pivot-shift grade 3.

Radiographs of the knee (coronal, sagittal) were performed before revision ACLR to evaluate bony structures such as PTS. The PTS was measured on the lateral knee radiograph and calculated by defining the angle between a line drawn tangentially to the tibial plateau and the proximal anatomic axis of the tibia [40]. A normal PTS was defined as 8 ± 3° [23].

Computed tomography (CT) with 3D reconstruction defined the tunnel position and bone tunnel enlargement that might suggest the need for a bone graft. Additionally, magnetic resonance imaging (MRI) of the injured knee was used to assess further lesions of the menisci, cartilage and other ligaments of the knee. Additionally, a standing long-leg radiograph to determine the mechanical axis of the lower leg was performed when valgus or varus deformity was clinically suspected. Valgus or varus deformity was defined when the mechanical femoro-tibial angle was more than 3°. The possible reasons for failure of primary ACLR are listed in Table 3.

### Surgical technique of revision ACLR

If the diameter of the bone tunnel was more than 12 mm or if a safe new tunnel position was not guaranteed due to the previous bone tunnel position, a two-stage revision procedure was performed. Bone tunnel filling was performed using allogeneic spongiosa. At the earliest 4 months after bone tunnel filling, revision ACLR was carried out. In the case of varus or valgus deformity with additional medial or lateral knee instability grade 2 or in the case of symptomatic unicompartmental osteoarthritis or varus or valgus deformity, the leg axis was corrected by osteotomy.

When a PTS ≥ 12° and no hyperextension of the knee joint occurred, slope-reducing osteotomy was performed in addition to bone tunnel filling (*n* = 5). Osteotomy of the tuberosity was performed via a skin incision 4–6 cm medial to the tibial tuberosity, creating a 7–10-cm-long tuberosity fragment connected to the patellar tendon. After prior computer-assisted planning, ventral closing-wedge high tibial osteotomy was performed at the mid-height of the tuberosity with the hinge point at the level of the tibial insertion of the posterior cruciate ligament. The osteotomy was fixed by lag screws using the tuberosity fragment as a "bioplate".

All revision ACLR procedures were performed with autografts during single-bundle ACLR using hamstring, bone–patellar–tendon–bone (BPTB) or quadriceps grafts. We attempted to preserve the meniscus. Peripheral meniscal lesions that could be repaired were sutured. When meniscal tears could not be repaired, partial meniscectomy was carried out.

Extra-articular lateral tenodesis using the modified “Lemaire” procedure was performed in 51 patients when high-grade anterior knee instability (side-to-side difference ≥ 6 mm and/or pivot-shift grade 3) and Lachman grade 3 occurred [42]. Via a 4-cm skin incision placed over the lateral epicondyle, a 6–8-cm-long and 6–8-mm-wide strip of the distal tractus was prepared with preserved connection to Gerdy’s tubercle. The free end was sewn with Vicryl suture, and a 2.4-mm K-wire was inserted into the lateral femur approximately 1 cm proximal and posterior to the lateral epicondyle. If the isometric examination showed slight tension of the tenodesis in extension, a 5–6 mm drill channel was placed over the 2.4-mm wire, and tenodesis was performed at 45°.

The Hughston technique was carried out (*n* = 22) when patients showed grade 2 medial knee instability in full extension or 30° flexion of the knee. Postero-medial reconstruction was performed according to the description of Jacobson et al. [21]. A 4–6 cm longitudinal incision of the skin was placed over the medial epicondyle and adductor tubercle. After splitting the sartorius fascia, the proximal superficial medial collateral ligament (sMCL) and posterior oblique ligament (POL) were demonstrated. In the interval between the sMCL and POL, the joint was opened longitudinally, and the deep MCL (dMCL) and the medial meniscus were revealed. The deep structures and dMCL were tightened with sutures, and any lesion of the postero-medial meniscus complex was reconstructed and sutured to the dMCL. Using a pants-over-vest suture technique, the POL was also sutured. Any possible damage to the semimembranosus insertion was reconstructed, and the sMCL was tensioned and closed.

When grade 3 medial knee instability was diagnosed, MCL reconstruction with an autologous graft was performed (*n* = 3). MCL reconstruction was performed according to the descriptions of Preiss et al. [31]. A 6-mm femoral bone tunnel was created at the intersection of an imaginary extension of the posterior edge of the tibia and the Blumensaat line via a 1–2 cm skin insertion under strict fluoroscopic control of the lateral knee. A gracilis tendon autograft sutured with Vicryl at both ends was placed in the femoral tunnel and fixed with an interference screw. Thus, one branch of the femoral tunnel was used for the sMCL and the second for the POL reconstruction. A 5-mm bone tunnel was created above the pes anserinus centrally to the sMCL insertion and above the insertion of the distal arm of the semimembranosus muscle. The sMCL and POL branches were inserted into the tibial bone tunnels below the sartorius fascia and fixed with interference screws at 30° of knee flexion.

### Statistical analysis

The calculation was based on two groups: stable revision ACLR and failed revision ACLR (recurrent instability after revision ACL surgery). Mean differences between treatment success at follow-up and treatment failure were calculated with unpaired Student's *t*-tests for parametric and Kruskal–Wallis tests for non-parametric parameters. Categorical parameters were compared using the Chi-square test. Additionally, multivariable conditional logistic regression analysis was performed to identify predictors for failure of revision ACLR, including body mass index (BMI), PTS, concomitant ligamentous injuries and meniscal lesions. A post hoc power analysis was performed using G*Power 3.1.9.6 for Mac to assess the validity of the proportions of medial instability in patients with successful and failed revision ACLR procedures.

Statistical analysis was performed using IBM® SPSS® Statistics version 22. A *p*-value less than 0.05 was considered significant.

## Results

### Demographic data and clinical findings before revision ACLR

The demographic data and preoperative clinical findings are summarized in Tables 4 and 5. There were 111 patients who were clinically evaluated after revision ACLR with a mean follow-up of 35 ± 3.4 months (24–67 months). Among them, 16 patients (14.5%) were identified with failure of revision ACLR. Based on our proportions of medial instability of 0.63/0.27 in 111 patients with 16 revision ACLR failures, a post hoc power of 0.868 was achieved at a significance level of 5%.

**Table 3:** Possible causes of primary ACLR failure

	Number of patients, *n* (%)
Femoral tunnel malposition	26 (23.4)
Tibial tunnel malposition	13 (11.7)
Trauma	18 (16.2)
Missed concomitant injury	54 (48.6)
More than one possible cause	67 (60.4)

**Table 4:** Demographic data of patients with revision ACLR (*n* = 111), *n.s.* = not significant, *STD* standard deviation

Characteristics	In total (*n* = 111)	Failed revision ACLR (*n* = 16)	Stable revision ACLR (*n* = 95)	*p* value
Female Sex, *n* (%)	43 (38.7)	6 (37.5)	37 (38.9)	*n.s*
Patient age, mean ± STD	31.7 ± 11 (15–58)	31.8 ± 10 (20–52)	31.7 ± 11.2 (15–58)	
Affected knee, left, *n* (%)	50 (45)	5 (31.3)	45 (47.4)	*n.s*
Body mass index (BMI) > 30 kg/m^2^, *n* (%)	30 (27)	12 (75)	18 (18.9)	*p* < 0.001
Two-stage revision ACLR with bone tunnel filling, *n* (%)	49 (44.1)	5 (31.3)	44 (46.3)	*n.s*
High-grade anterior knee laxity, *n* (%)	41 (36.9)	11 (68.8)	30 (31.6)	*p* = 0.004
Number of previous ACLR procedures, *n* (%)				
1 previous ACLR procedure	94 (85.5)	12 (75)	82 (87.2)	*n.s*
2 previous ACLR procedures	13 (11.8)	4 (25)	9 (9.6)	
3 previous ACLR procedures	1 (0.9)	0	1 (1.1)	
4 previous ACLR procedures	2 (1.8)	0	2 (2.1)	
Time between revision ACLR and previous ACLR in months, mean ± SD	47.5 ± 37.6 (12–181)	45.3 ± 44.3 (12–178)	47.9 ± 36.6 (14–181)	*n.s*
Traumatic reinjury after revision ACLR *n* (%)	1 (0.9)	1 (6.3)	0	–

**Table 5:** Clinical findings before revision ACLR (*n* = 111), *n.s.* = not significant, *STD* standard deviation

Characteristics	In total (*n* = 111)	Failed revisio*n* ACLR (n = 16)	Stable revisio*n* ACLR (n = 95)	*p* value
Grade of Lachman test preoperatively, *n* (%)				
Grade 1 (2–5 mm)	29 (26.1)	4 (25)	25 (26.3)	*p* = 0.05
Grade 2 (5–10 mm)	72 (64.9)	8 (50)	64 (67.4)	
Grade 3 (> 10 mm)	10 (9)	4 (25)	6 (6.3)	
Rolimeter side-to-side difference preoperatively, mean in mm ± STD (minimum—maximum)	6 ± 2.4 (3–13)	7 ± 3.2 (4–13)	5.9 ± 2 (3–10)	*p* < 0.001
Grade of pivot-shift test preoperatively, *n* (%)				
Grade 1 (glide)	19 (17.1)	1 (6.3)	18 (18.9)	n.s
Grade 2 (clunk)	59 (53.2)	11 (68.8)	48 (50.5)	
Grade 3 (gross)	28 (25.2)	4 (25)	24 (25.3)	
Lateral knee instability preoperatively, *n* (%)	18 (16.2)	5 (31.3)	13 (13.7)	n.s
Lateral knee instability grade 1	18 (16.2)	5 (31.3)	13 (13.7)	n.s
Lateral knee instability grade 2	0	0	0	
Lateral knee instability grade 3	0	0	0	
Medial knee instability preoperatively, *n* (%)	36 (32.4)	10 (62.5)	26 (27.4)	*p* = 0.005
Medial knee instability grade 1	11 (9.9)	5 (31.3)	6 (6.3)	*p* = 0.05
Medial knee instability grade 2	22 (19.8)	4 (25)	18 (18.9)	
Medial knee instability grade 3	3 (2.7)	1 (6,3)	2 (2.1)	
Long leg standing axis, *n* (%)				
Normal long leg standing axis	101 (91)	14 (87.5)	87 (91.6)	*n.s*
Valgus deformity (> 3°)	3 (2.7)	0	3 (3.2)	
Varus deformity (> 3°)	7 (6.3)	2 (12.5)	5 (5.3)	
PTS greater than 11°, *n* (%)	15 (13.5)	6 (37.5)	9 (9.5)	*p* = 0.002
Femoral tunnel width preoperatively, mean ± STD	9.92 ± 2.8 (3–21)	8.9 ± 2.8 (5–15)	10.1 ± 2.8 (3–21)	*n.s*
Tibial tunnel width preoperatively, mean ± STD	10.4 ± 3.3 (0–18)	9.9 ± 2.3 (6–14)	10.5 ± 3.4 (0–18)	*n.s*

**Fig. 1:**
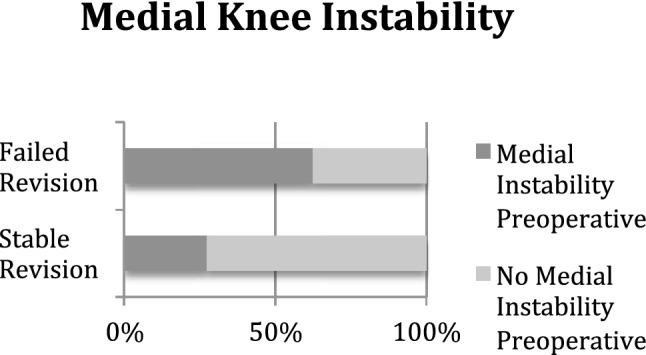
Preoperative medial knee instability occurred significantly more often in patients with failed revision ACLR than in patients with stable revision ACLR

Preoperative medial knee instability (before revision ACLR) was significantly more frequent in the failed surgery group than in the stable revision ACLR group (63% vs. 27%; *p* = 0.005) (Fig. 1).

**Fig. 2:**
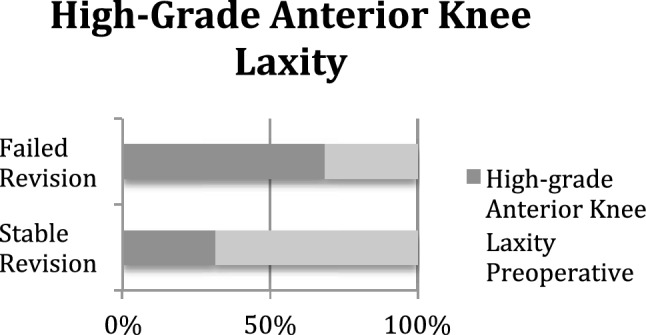
Preoperative high-grade anterior knee laxity was found significantly more often in the failed revision ACLR group than in the stable revision ACLR group

**Fig. 3:**
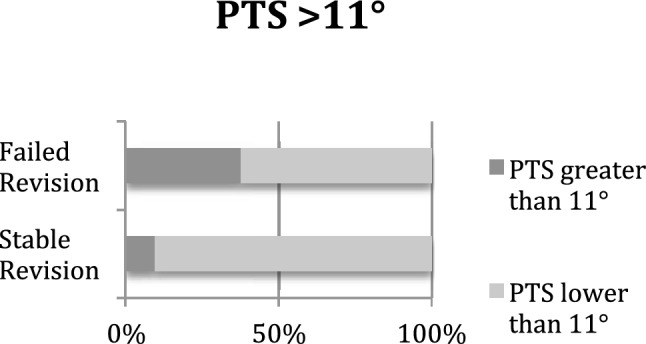
Patients with failed revision ACLR significantly more often showed an elevated PTS greater than 11° than patients with stable revision ACLR

**Fig. 4:**
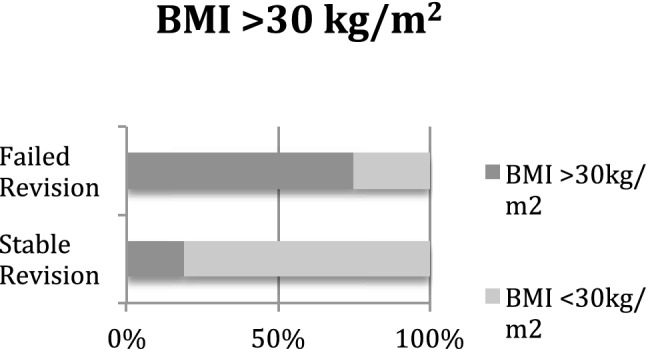
Patients were significantly more often obese in the failed revision ACLR group than in the stable revision ACLR group

High-grade anterior knee laxity occurred significantly more often in the failed revision ACLR group (69% vs. 32%; *p* = 0.004) (Fig. 2). Additionally, the percentage of patients with an increased PTS was significantly larger in the failed revision ACLR group than in the other group (38% vs. 10%; *p* = 0.002) (Fig. 3). While patients with failed revision ACLR showed significantly higher anterior translation of the tibia preoperatively in the third grade Lachman test (25% vs. 6%; *p* = 0.05) and a significantly greater Rolimeter® side-to-side difference (7 mm vs. 5.9 mm; *p* < 0.001), they were also significantly more often obese with a BMI of greater than 30 kg/m^2^ (75% vs. 19%; *p* < 0.001) (Fig. 4) and demonstrated more medial meniscus lesions (81% vs. 40%; *p* = 0.002). The meniscus status at the time of revision ACLR, the revision graft choice and additional procedures during revision ACLR are displayed in Tables 6 and 7. Valgus-opening high tibial osteotomy in cases of varus deformity and additional symptomatic medial osteoarthritis was performed in seven patients, and slope-reducing high tibial osteotomy combined with bone tunnel filling was performed in five patients. Leg axis correction osteotomies were not performed for medial or lateral knee instability in combination with leg axis deformity (Table 7).

**Table 6:** Meniscal status at the time of revision ACLR (*n* = 111), *n.s. = *not significant

Characteristics	In total (*n* = 111)	Failed revision ACLR (*n* = 16)	Stable revision ACLR (*n* = 95)	*p* value
Medial meniscal lesion in total, *n* (%)	51 (45.9)	13 (81.3)	38 (40)	*p* = 0.002
Medial meniscus repair, *n* (%)	38 (34.2)	9 (56.3)	29 (30.5)	*p* = 0.002
Partial medial meniscus resection, *n* (%)	12 (10.8)	3 (18.8)	9 (9.5)	
Total medial meniscus resection, *n* (%)	0	0	0	
Medial meniscus transplantation, *n* (%)	1 (0.9)	1 (6.3)	0	
Lateral meniscal lesion in total, *n* (%)	23 (20.7)	1 (6.3)	22 (23.2)	*n.s*
Lateral meniscus repair, *n* (%)	16 (14.4)	0	16 (16.8)	*n.s*
Partial lateral meniscus resection, *n* (%)	7 (6.3)	1 (6.3)	6 (6.3)	
Total lateral meniscus resection, *n* (%)	0	0	0	
Lateral meniscus transplantation, *n* (%)	0	0	0	

### Predictors for failure of revision ACLR

The results of multivariable conditional logistic regression analysis for predictors are displayed in Table 8. Preoperative medial knee instability (odds ratio, 16.8 [95% CI, 1.7–164]; *p* = 0.015), preoperative high-grade anterior knee laxity (odds ratio, 4.9 [95% CI, 1.1–21.6]; *p* = 0.034), an abnormal PTS (odds ratio, 4.9 [95% CI, 1–36.9]; *p* = 0.046) and a BMI greater than 30 kg/m^2^ (odds ratio, 8.6 [95% CI, 1.8–42.1]; *p* = 0.008) were determined to be independent risk factors associated with failure of revision ACLR.

**Table 7:** Revision ACLR graft choice and additional procedures during revision ACLR (*n* = 111), *n.s.* = not significant, *BTPB* = bone–tendon–patellar–bone, *HTO* = high tibial osteotomy

Characteristics	In total (*n* = 111)	Failed revision ACLR (*n* = 16)	Stable revision ACLR (*n* = 95)	*p* value
Choice of revision ACLR graft, *n* (%)				
BTPB graft	64 (57.7)	12 (75)	52 (54.7)	*n.s*
Ipsilateral hamstring graft	20 (18)	2 (12.5)	18 (18.9)	
Contralateral hamstring graft	12 (10.8)	1 (6.3)	11 (11.6)	
Quadriceps graft	15 (13.5)	1 (6.3)	14 (14.7)	
Medial stabilized operatively in total, *n* (%)	25 (22.5)	5 (31.3)	20 (21,1)	*n.s*
Houghston, *n* (%)	22 (19.8)	4 (25)	18 (18.9)	*n.s*
MCL graft reconstruction, *n* (%)	3 (2.7)	1 (6.3)	2 (2.1)	*n.s*
Extra-articular lateral tenodesis, *n* (%)	51 (45.9)	8 (50)	43 (45.3)	*n.s*
Valgus-open HTO	7 (6.3)	1 (6.3)	6 (6.3)	*n.s*
Slope reduction osteotomy	5 (4.5)	0	5 (5.3)	*n.s*

**Table 8:** Logistic regression model for predictors of failure of revision ACLR, *n.s.* = not significant

Characteristics	Odds ratio (95% CI)	*p* value
Medial knee instability preoperatively	16.8 (1.7–164) Reference (1.0)	*p* = 0.015
Lateral knee instability preoperatively	0.88 (0.1–7.8) Reference (1.0)	*n.s*
High-grade anterior knee laxity preoperatively	4.9 (1.1–21.6) Reference (1.0)	*p* = 0.034
BMI greater than 30 kg/m^2^ preoperatively	8.6 (1.8-42.1) Reference (1.0)	*p* = 0.008
PTS greater than 11° preoperatively	6.2 (1–36.9) Reference (1.0)	*p* = 0.046
Medial stabilized during revision ACLR	– 13.3 (1.2–146.2) Reference (1.0)	*p* = 0.034
Lateral extra-articular tenodesis during revision ACLR	– 0.38 (0.8–1.7) Reference (1.0)	*p* = 0.028

### Factors reducing the failure risk of revision ACLR

The multivariable conditional logistic regression analysis showed a reduced risk for failure of revision ACLR when additional medial (odds ratio,  – 13.3 [95% CI, 1.2–146.2]; *p* = 0.034) and/or anterolateral (odds ratio,  – 1.5 [95% CI, 0.8–1.7]; *p* = 0.028) stabilization was performed at the time of revision surgery.

## Discussion

The most important finding of this study was that preoperative medial knee instability is a risk factor for revision ACLR and should be adequately addressed at the time of revision ACLR. This study demonstrates the largest revision ACLR patient group with pre- and postoperative clinical examination data and a follow-up of 2 years published to date and it indicates that preoperative knee instability is an important factor for the treatment strategy of revision ACLR. Medial knee instability, high-grade anterior knee instability and increased PTS are risk factors for failure of revision ACLR and should be addressed at the time of revision surgery. Additionally, a high BMI is a risk factor for revision ACLR.

Concomitant instability is reported to be a risk factor for failure of primary ACLR, as it can lead to more knee instability and potentially place more strain on the ACL [22, 28, 35]. O’Brien et al. demonstrated in their study that all patients with knee instability after ACLR had concomitant ligamentous instability that was not detected or addressed at the time of ACLR [29].

In this study, failure of revision ACLR was associated with medial knee instability on preoperative examination. In the literature, this result has not yet been described for revision ACLR.

The medial and postero-medial structures (functional complex of the MCL, posterior oblique ligament (POL), postero-medial capsule and medial meniscus) are important secondary stabilizers, especially against rotation of the knee [14, 17]. A partial or complete MCL lesion increases the load on the ACL depending on the knee flexion angle for anterior torque up to 20% and for valgus torque up to 185% [3].

Interestingly, in this study, accompanying medial knee instability led to a 17 times greater risk of failure of revision ACLR when preoperatively presented. Undiagnosed injury to the medial collateral ligament will lead to increased translational and rotational instability, which can also be the reason for primary ACLR failure. For primary ACLR in cases of grade 2 medial instability, a 13-fold increased risk of failure was reported [1].

The treatment strategies for chronic medial instability in combination with primary ACL insufficiency are not consistent in the current literature. Zaffagnini et al. compared lesions of chronic MCL and primary ACL ruptures (treated with ACLR and conservative therapy of the MCL) with isolated ACL ruptures (treated with ACLR). Except for persistent medial instability without relevant functional limitations, there was no relevant clinical difference in their study population [45]. In contrast, Funchal et al. showed in their prospective randomized controlled study significantly lower failure rates and better clinical results when patients with combined chronic MCL and primary ACL ruptures were treated with ACLR and medial anatomical reconstruction [11]. Additionally, Svantesson et al. demonstrated an increased risk of ACL revision with non-surgical treatment of a concomitant medial collateral ligament injury in their register trial of 19,457 patients [37]. The findings of the presented analysis support these studies, as chronic medial instability, especially grade 2 and higher in combination with revision ACLR, should be surgically addressed at the time of revision surgery.

The strategy of treating grade 2 medial knee instability with augmented repair (Hughston) and grade 3 medial knee instability with anatomical reconstruction is based on biomechanical data by Widicks et al., who were able to show in a cadaver model that both procedures were equally capable of restoring knee joint stability [41]. In the present study, surgical treatment of the medial side reduced the risk of failure of revision ACLR by a factor of 13. Nevertheless, this study showed a failure rate for augmented repair of the medial side in 18% (4 out of 22 augmented repairs) of the cases, and the results seem to be worse than the clinical results of augmented repairs in combination with primary ACLR in the literature [6, 8, 30].

The good clinical results of anatomical medial reconstruction with tendon grafts, for example, in studies by Funchal et al. and Lind et al. in combined MCL and primary ACLR, as well as the better objective knee joint stability achieved by Dong et al. in anatomical reconstruction compared to repair, suggest that anatomical medial reconstruction with tendon grafts may be superior to repair [11, 24]. However, the number of cases of anatomical reconstruction in this study is too small to prove that anatomical reconstruction with tendon grafts is associated with lower failure rates than the Hughston repair technique.

In recent years, the value of peripheral structures, especially the ALL, has been more of a focus of ACLR [34]. Louis et al. reported a very low failure rate of 1.2% in revision ACLR with additional ALL stabilization (7). However, 13.5% of the patients showed a persistent instability of greater than 5 mm from instrumental measurements [25]. In this study, a side-to-side difference greater than 5 mm was defined as failed revision ACLR. Nevertheless, in line with Louis et al., this study showed a reduced risk of recurrent revision of ACLR when extra-articular lateral tenodesis was performed.

High-grade anterior knee laxity has been demonstrated to lead to an increased risk of revision in ACLR [26]. Heijne et al. demonstrated the correlation between increased preoperative knee laxity and lower KOOS quality of life scores 1 year postoperatively [15]. When high-grade anterior knee laxity occurred preoperatively, the patients in the present study had a 5 times greater risk of failure of revision ACLR. The authors have suggested that additional augmentation procedures control high-grade anterior knee laxity and improve outcomes [33]. The findings of this study correlate with these results, as patients in this study population had a reduced risk of failure of revision ACLR when extra-articular lateral tenodesis was performed during revision ACLR, proving that high-grade anterior instability, especially in revision ACLR, is an indication for lateral extra-articular tenodesis.

An increased PTS is associated with a high risk of failure of ACLR, as it can lead to an anterior shift of the tibia [13]. Bernhardson et al. found that the ACL graft force in the loaded testing state increased linearly as the slope increased [4]. This study showed that a higher risk of revision ACLR failure was associated with an elevated preoperative PTS. This study proved, in line with other studies, that an increased PTS can be a risk factor for recurrent ACLR injuries [13, 46].

Obesity was detected to be a risk factor for failure after revision ACLR in the population in the presented study. Thus, patients had a 9 times greater risk of failure when they were obese (BMI ≥ 30 kg/m^2^). Previous studies also demonstrated a correlation between elevated BMI and the risk of ACL injuries through a non-contact mechanism [16, 36, 39]. According to the results presented here, obesity is associated with an increased rate of re-rupture after revision ACLR.

The accurate diagnosis of rotational instabilities can be challenging. As validated tests for the classification of rotational instabilities do not exist, these instabilities could not be clearly detected in this study. Medial and lateral knee instability is often combined with rotational instability of the knee. A high degree of anterior knee instability combined with third grade pivot-shift and positive dial-test provide evidence for rotational instability, but validated clinical tests that clearly classify an insufficiency of the anterolateral or postero-medial structures do not yet exist.

## Conclusions

This study demonstrates the largest revision ACLR patient group with pre- and postoperative clinical examination data and a follow-up of 2 years published to date. Preoperative medial knee instability is an underestimated risk factor for revision ACLR failure.

Additionally, high-grade anterior knee laxity, increased PTS and elevated BMI had an elevated risk for failure after revision ACLR, while medial stabilization and lateral extra-articular tenodesis decreased the likelihood of revision graft failure.
